# Drug Repurposing for the Treatment of Bacterial and Fungal Infections

**DOI:** 10.3389/fmicb.2019.00041

**Published:** 2019-01-28

**Authors:** Andrea Miró-Canturri, Rafael Ayerbe-Algaba, Younes Smani

**Affiliations:** Clinical Unit of Infectious Diseases, Microbiology and Preventive Medicine, Institute of Biomedicine of Seville (IBiS), University Hospital Virgen del Rocío, CSIC, University of Seville, Seville, Spain

**Keywords:** repurposing drug, bacteria, fungi, antimicrobial resistance, infection

## Abstract

Multidrug-resistant (MDR) pathogens pose a well-recognized global health threat that demands effective solutions; the situation is deemed a global priority by the World Health Organization and the European Centre for Disease Prevention and Control. Therefore, the development of new antimicrobial therapeutic strategies requires immediate attention to avoid the ten million deaths predicted to occur by 2050 as a result of MDR bacteria. The repurposing of drugs as therapeutic alternatives for infections has recently gained renewed interest. As drugs approved by the United States Food and Drug Administration, information about their pharmacological characteristics in preclinical and clinical trials is available. Therefore, the time and economic costs required to evaluate these drugs for other therapeutic applications, such as the treatment of bacterial and fungal infections, are mitigated. The goal of this review is to provide an overview of the scientific evidence on potential non-antimicrobial drugs targeting bacteria and fungi. In particular, we aim to: (i) list the approved drugs identified in drug screens as potential alternative treatments for infections caused by MDR pathogens; (ii) review their mechanisms of action against bacteria and fungi; and (iii) summarize the outcome of preclinical and clinical trials investigating approved drugs that target these pathogens.

## Introduction

Bacteria and fungi are highly efficient in acquiring antimicrobial resistance encoded by genomic changes ranging in scale from point mutations, through the assembly of preexisting genetic elements, to the horizontal import of genes from the environment (Kung et al., 2010; Cowen et al., 2015; Yelin and Kishony, 2018). Compounding the problem of antimicrobial resistance is the immediate threat of a reduction in the discovery and development of new antibiotics, the dangers of which have recently been made clear by the World Health Organization (WHO) (Tacconelli et al., 2018) and other European institutions (O’Neill, 2016; Årdal et al., 2018). Consequently, a perfect storm is converging with regard to these infections: increasing antimicrobial resistance with a decreased new drug development (O’Neill, 2016). This context is likely the best example of the purported “Post-Antibiotic Era,” with relevance even in non-specialized media (Bagley and Outterson, 2017). It is clear that effective solutions are urgently needed as stressed by various institutions.

New policies and actions are necessary to avoid the figures predicted for 2050 that attribute ten million deaths worldwide to antimicrobial resistance (O’Neill, 2016). Such efforts might include: a massive global public awareness campaign; improvements in hygiene and prevention of the spread of infection; increase global surveillance of drug resistance and the appropriate antimicrobial consumption in humans and animals; the promotion of novel rapid diagnostics to curtail the unnecessary use of antimicrobial agents; and the promotion, development, and use of vaccines and other alternatives to both prevent and treat bacterial infections (O’Neill, 2016). Therefore, the development of new antimicrobial therapeutic strategies for use alone or together with one of the scarce but clinically relevant antibiotics has become exigent. In this environment, “repurposing” (defined as investigating new uses for existing drugs) has gained renewed interest, as reflected by several recent studies (Fischbach and Walsh, 2009; Brown, 2015; Rampioni et al., 2017). The combination of these existing drugs with antimicrobial agents currently in clinical use is also under consideration.

A literature review was conducted to search for potential non-antimicrobial candidate drugs that are not intentionally used as antimicrobial agents but have one or more antimicrobial properties. A variety of drug families have been considered including: anthelmintics (Lim et al., 2013; Rajamuthiah et al., 2015; Gooyit and Janda, 2016; Joffe et al., 2017); anticancer drugs (Ueda et al., 2009; Butts et al., 2014; Yeo et al., 2018); anti-inflammatory/immunomodulatory drugs (Artini et al., 2014; Thangamani et al., 2015b; Ogundeji et al., 2016); antipsychotic and antidepressant drugs (Lieberman and Higgins, 2009; Andersson et al., 2016; Holbrook et al., 2017); statins (Parihar et al., 2014; Thangamani et al., 2015a; Ribeiro et al., 2017); and iron-storage drugs (Gi et al., 2014). While these drugs are approved for their clinical indications, promising antibacterial and antifungal activities have been reported in preclinical and clinical studies. It is noteworthy that repurposing drugs is a very promising approach with several advantages. As drugs approved by the Federal Drug Administration (FDA), information about their pharmacological characteristics (both safety and pharmacokinetic) in preclinical and clinical trials is widely available. Therefore, the time and economic costs associated with the repurposing of these drugs for other therapeutic applications such as the treatment of bacterial and fungal infections will be minimized.

In this review, we focus on the current state of knowledge regarding the repurposing of drugs in terms of their modes of action, antimicrobial efficacy and breadth of spectrum against bacteria and fungi, as well as the advances to-date in their development as antimicrobial agents for clinical use. To this end, we introduce in Pubmed database different key words such as repurposing drugs, antibacterial and/or antifungal in order to find published literature about the repurposing drugs for treatment of bacterial and fungal infections.

## Potential Drugs for Repurposing Against Infectious Agents

**FIGURE 1:**
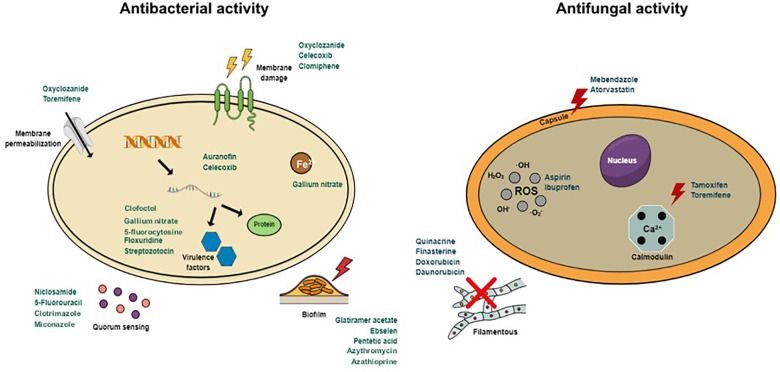
Antibacterial and antifungal mechanisms of action of repurposed drugs.

The antibacterial and antifungal activities of repurposing drugs and their modes of action are summarized in Table 1 and Figure 1.

**Table 1:** Direct antibacterial and antifungal activity of repurposed drugs.

Drugs	Clinical indication	Target bacteria	Mechanisms of action	Reference
Niclosamide^∗^	Helminthiasis	*P. aeruginosa*	Inhibition of quorum sensing and various virulence genes, and reduction of elastase and pyocyanin levels	Imperi et al., 2013b
Oxyclozanide	Helminthiasis	*S. aureus*	Bacterial membrane damage	Rajamuthiah et al., 2015
Mebendazole	Helminthiasis	*C. neoformans*	Morphological alterations by reducing capsular dimensions	Joffe et al., 2017
Quinacrine	Helminthiasis	*C. albicans*	Inhibition of filamentation	Kulkarny et al., 2014
Floxuridine	Colorectal cancer	*S. aureus*	Inhibition of the SaeRS two-component system, and inhibition of the transcription of other virulence regulatory systems	Yeo et al., 2018
Streptozotocin	Pancreatic islet cell cancer	*S. aureus*	Inhibition of the SaeRS two-component system, and inhibition of the transcription of other virulence regulatory systems	Yeo et al., 2018
Toremifene	Breast cancer	*S. mutans* and *P. gingivalis*	Membrane permeabilization and damage	Gerits et al., 2017
		*C. neoformans*	Binding to the two essential EF-hand proteins calmodulin 1 (Cam1) and calmodulin-like protein (Cml) and prevention of Cam1 from binding to its well-characterized substrate calcineurin	Butts et al., 2014
Tamoxifen	Breast cancer	*C. neoformans*	Binding to the two essential EF-hand proteins calmodulin (Cam1) and calmodulin-like protein (Cml) and prevention of Cam1 from binding to its well-characterized substrate calcineurin	Butts et al., 2014
Raloxifene	Breast cancer	*P. aeruginosa*	Binding to PhzB2 which is involved in the production of pyocyanin, a pigment related with the virulence of *P. aeruginosa*	Ho Sui et al., 2012
Clomiphene	Fertility	*S. aureus*	Inhibition of undecaprenyl diphosphate synthase involved in the synthesis of teichoic acid wall	Farha et al., 2015
Finasteride	Benign prostatic hyperplasia	*C. albicans*	Inhibition of filamentation	Routh et al., 2013
5-fluorouracil	Solid tumors	*P. aeruginosa*	Inhibition of biofilm formation and quorum sensing	Ueda et al., 2009
Doxorubicin	Bladder, breast, stomach, lung, ovarian, and thyroid cancers	*C. albicans*	Inhibition of filamentation	Chavez-Dozal et al., 2014
Daunorubicin	Acute myeloid leukemia, acute lymphocytic leukemia, chronic myelogenous leukemia, and Kaposi’s sarcoma	*C. albicans*	Inhibition of filamentation	Chavez-Dozal et al., 2014
Clofoctol	Bacterial infection	*P. aeruginosa*	Inhibition of the pqs system, probably by targeting the transcriptional regulator PqsR	D’Angelo et al., 2018
Azithromycin	Bacterial infection	*P. aeruginosa*	Interaction with the ribosome, resulting in direct and/or indirect repression of specific subsets of genes involved in virulence, quorum sensing, biofilm formation,	Imperi et al., 2014
5-fluorocytosine	Fungal infection	*P. aeruginosa*	Inhibition of the production of pyoverdine, Prlp protease, and exotoxin A by downregulation of the *pvdS* gene.	Imperi et al., 2013a
Clotrimazole and miconazole	Fungal infection	*P. aeruginosa*	Inhibition of the *pqs* activity through the possible inactivation of 2-alkyl-4-quinolones (AQ) production or reception	D’Angelo et al., 2018
Gallium nitrate^∗^	Lymphoma and bladder cancer	*P. aeruginosa*	Effects on iron metabolism	Antunes et al., 2012
Celecoxib	Inflammation	*S. aureus, B. anthracis, B. subtilis*, and *M. smegmatis*	Inhibition of bacterial DNA, RNA, protein synthesis, and cell wall	Thangamani et al., 2015b
Diflunisal	Inflammation	*S. aureus*	Inhibition of ArgA, a regulator protein which inhibits alpha-type phenol soluble modulins toxins	Hendrix et al., 2016
Glatiramer acetate	Inflammation	*P. aeruginosa*	Disruption of biofilm formation	Christiansen et al., 2017
Aspirin and ibuprofen	Inflammation	*C. neoformans* and *C. gattii*	Stress induction via activation of the high-osmolarity glycerol (HOG) pathway, and activation of reactive oxygen species (ROS)-mediated membrane damage	Ogundeji et al., 2016
Pimozide	Severe Tourette’s syndrome and schizophrenia	*L. monocytogenes*	Reduction of *L. monocytogenes* internalization by phagocytic cells by decreasing vacuolar escape and diminishing cell-to-cell spread	Lieberman and Higgins, 2009
Azathioprine	Crohn’s disease	*P. aeruginosa* and *E. coli*	Inhibition of WspR. WspR is a diguanylate cyclase involved in the regulation of a signal molecule called cyclic-di-GMP (c-di-GMP) known as a regulated of the bacterial biofilm formation	Antoniani et al., 2013
Simvastatin	Hypercholesterolemia	*M. tuberculosis*	Reduction of cholesterol within phagosomal membrane	Parihar et al., 2014
Atorvastatin	Hypercholesterolemia	*C. gattii*	Reduction of the ergosterol content in the cell membrane and alteration of the properties of the polysaccharide capsule; increase in the production of ROS by macrophages; and reduction of yeast phagocytosis and the intracellular proliferation rate	Ribeiro et al., 2017
Ebselen^∗^	Bipolar disorder and ischemic stroke	*S. aureus, C. difficile*	Reduction of biofilm formation and targeting of the glucosyltransferase domain toxins	Gi et al., 2014; Peng et al., 2018
Pentetic acid	Hypocalcemia	*P. aeruginosa*	Reduction of biofilm formation and inhibition of elastase	Gi et al., 2014
Auranofin	Rheumatoid arthritis	*S. aureus*	DNA inhibition and protein synthesis, and downregulation of toxin production	Thangamani et al., 2016a


*^∗^ Some of the drugs listed in this table displayed multiple effects against different pathogens, and that only the findings of an exemplifying study are reported in the table. Other studies on the same drugs are discussed in the text.*

### Anthelmintic Drugs Repurposed Against Bacteria and Fungi

Anthelmintic drugs constitute a large class of medications used for the treatment of helminthiasis. Their activities aside from their use against parasitic infections are being investigated in other areas such as oncology (Dogra et al., 2018; Wang et al., 2018). The activity of these drugs against Gram-positive and Gram-negative bacteria, and fungi has been reported. The salicylanilide family contains a number of the anthelmintic drugs approved for the treatment of parasitic infections. The most widely used members of this family include niclosamide for humans (Chen et al., 2018) and oxyclozanide, rafoxanide, and closantel for animals (Martin, 1997).

The mode of action of salicylanilides is not completely understood, but they are thought to act as uncouplers of oxidative phosphorylation, thereby impairing the motility of parasites. Rajamuthiah et al. (2015) described the efficacy of niclosamide and oxyclozanide against methicillin-, vancomycin-, linezolid-, or daptomycin-resistant *Staphylococcus aureus* isolates. They reported that niclosamide presented bacteriostatic activity whereas oxyclozanide exhibited antibacterial action, likely due to damage in the bacterial membrane. Together with niclosamide and oxyclozanide, other members of the salicylanilides family such as rafoxanide and closantel have presented greater bactericidal activity against the logarithmic and stationary phases of *Clostridium difficile* than vancomycin (Gooyit and Janda, 2016). Avermectins, a broad-spectrum class of anthelmintic drugs which include ivermectin, selamectin, and moxidectin, demonstrated efficacy *in vitro* against *Mycobacterium tuberculosis* and *Mycobacterium ulcerans* with minimum inhibitory concentration (MIC) values ranging from 1 to 8 mg/L and 4 to 8 mg/L, respectively (Lim et al., 2013; Omansen et al., 2015). Moreover, ivermectin showed efficacy against *S. aureus* clinical isolates including methicillin-resistant strains (MRSA) (Ashraf et al., 2018). *In vivo*, ivermectin improves LPS-induced survival in mice by reducing serum and murine macrophage levels of TNF-α, IL-1β, and IL-6 and blocking the NF-*κ*B pathway (Zhang et al., 2008).

In Gram-negative bacteria, only niclosamide exhibited antibacterial activity. This drug showed an anti-virulent effect against *Pseudomonas aeruginosa* via the inhibition of quorum sensing and virulence genes, reducing elastase and pyocyanin levels (Imperi et al., 2013b). In *Acinetobacter baumannii* and *Klebsiella pneumoniae*, niclosamide is able to increase the proportion of negative charges on their cell walls, and to potentiate the activity of colistin against colistin-resistant *A. baumannii* and *K. pneumoniae* (Ayerbe-Algaba et al., 2018). Recently, the effectiveness of niclosamide against *Helicobacter pylori* has been described, showing an MIC of 0.25 mg/L against the ATCC 49503 strain (Tharmalingam et al., 2018). Furthermore, niclosamide has demonstrated an immunomodulatory role by decreasing the secretion of IL-8 in a gastric cancer cell line after *H. pylori* infection (Tharmalingam et al., 2018). Niclosamide also showed therapeutic efficacy in an experimental infection model of *Galleria mellonella* larvae infected with *P. aeruginosa* and *H. pylori* (Imperi et al., 2013b; Tharmalingam et al., 2018). The formulation of niclosamide under nanosuspension showed lower toxicity in a rat lung infection model involving *P. aeruginosa*; the results of this study are potentially favorable for the further study of this formulation (Costabile et al., 2015).

In the case of fungi, mebendazole inhibited the growth of *Cryptococcus neoformans* and *Cryptococcus gattii* and affected the formation of biofilm by *C. neoformans* (Joffe et al., 2017). The combination of mebendazole with amphotericin B increased the fungicidal activity of amphotericin B against *C. neoformans* twofold (Joffe et al., 2017). Moreover, quinacrine, in monotherapy, has been shown *in vitro* to be effective for the prevention and treatment of *Candida albicans* biofilms, accumulating in vacuoles and causing defects in endocytosis (Kulkarny et al., 2014). In combination with caspofungin or amphotericin B, quinacrine has demonstrated synergy against *C. albicans* (Kulkarny et al., 2014).

These studies highlight the potential use of the anthelmintic drugs as antimicrobial agents as monotherapy for infections caused by Gram-positive and Gram-negative bacteria and fungi; although *in vivo* studies in vertebrate experimental models should be conducted.

### Anticancer Drugs Repurposed Against Bacteria and Fungi

The antibacterial activity of anticancer drugs has also been reported (Soo et al., 2017). Most of them act against Gram-positive pathogens.

The FDA-approved anticancer drugs floxuridine (mostly used in colorectal cancer) and streptozotocin (used for pancreatic islet cell cancer) have exhibited activity against *S. aureus* by inhibiting the SaeRS two-component system (TCS) (Yeo et al., 2018). SaeRS TCS is an important transcriptional regulator of different virulence factors of *S. aureus* including adhesins, toxins, and enzymes (Yeo et al., 2018). Floxuridine showed direct antibacterial activity by inhibiting the growth of *S. aureus* USA300 at a concentration of 0.0625 mg/L *in vitro* and increasing the survival of mice by 60% in a murine model of blood infection *in vivo* (Yeo et al., 2018). On the other hand, streptozotocin did not affect staphylococcal growth *in vitro* but reduced the mortality of mice to 10% *in vivo* (Yeo et al., 2018). Both drugs not only cause significant changes in the transcription of *S. aureus* genes, but also inhibit the transcription of other virulence regulatory systems of *S. aureus* (Yeo et al., 2018).

Another group of anticancer drugs developed to combat breast cancer is the selective estrogen receptor modulators (SERMs). Tamoxifen has been reported to exhibit activity against *S. aureus* (Corriden et al., 2015) and its analog toremifene showed efficacy against oral infection caused by *Streptococcus mutans* (Gerits et al., 2017). Toremifene also has been shown to reduce biofilm formation by *S. mutans* due to a possible increase in membrane permeabilization and therefore, membrane damage (Gerits et al., 2017). Clomiphene, another SERM in preclinical development for the treatment of fertility, has demonstrated efficacy against *S. aureus* and *Bacillus subtilis in vitro*, with an MIC value of 8 mg/L, and incubation of *B. subtilis* with this concentration of clomiphene changed its morphology (Farha et al., 2015). The mode of action of clomiphene is through the inhibition of undecaprenyl diphosphate synthase (UPPS), an enzyme involved in the synthesis of the teichoic acid wall of *S. aureus* (Farha et al., 2015). Due to this action on the bacterial wall, clomiphene exhibits synergy with β-lactams in restoring MRSA susceptibility (Farha et al., 2015).

Other anticancer drugs were tested as adjunctive therapies against *M. tuberculosis* infection. One such drug, denileukin diftitox, is currently used for the treatment of cutaneous T-cell lymphoma (Gupta et al., 2017). Treatment with denileukin diftitox slightly reduced the lung bacterial count in mice aerosol-infected with *M. tuberculosis* (Gupta et al., 2017). The addition of this drug to standard tuberculosis treatments, composed of rifampin, isoniazid, and pyrazinamide, similarly reduced the bacterial burden (Gupta et al., 2017).

Different studies have been also performed on Gram-negative bacteria to evaluate the antibacterial effect of anticancer drugs. A potent anticancer drug indicated for the treatment of different types of solid tumors called 5-fluorouracil, as well as gallium nitrate, an anticancer drug for the treatment of lymphoma and bladder cancer, have been extensively studied (Banin et al., 2008; Bonchi et al., 2014; Minandri et al., 2014; Rangel-Vega et al., 2015). 5-fluorouracil has been used against a collection of 5,850 mutants of the *P. aeruginosa* PA14 strain, revealing positive activity via the regulation of a large number of genes involved in quorum sensing and biofilm formation (Ueda et al., 2009; Rangel-Vega et al., 2015). Also, gallium nitrate has demonstrated *in vitro* an inhibitory effect on bacterial growth in a collection of 58 multidrug-resistant (MDR) *A. baumannii* strains, and in *P. aeruginosa* at concentrations >3.13 μM (Kaneko et al., 2007; Antunes et al., 2012; Frangipani et al., 2014); although the presence of pyoverdine and proteases in human serum reduce the efficacy of gallium nitrate against *P. aeruginosa* by increasing its MIC (Bonchi et al., 2015). At non-bactericidal concentrations, gallium nitrate can affect the production of virulence factors of *P. aeruginosa* (Kaneko et al., 2007; García-Contreras et al., 2014). In *G. mellonella*, the administration of this drug alone or in combination with colistin, at concentrations mimicking the human therapeutic dose of gallium nitrate used for cancer patients (28 μM), significantly increased the survival of larvae after infection by *A. baumannii* (Antunes et al., 2012). Moreover, in murine models of acute and chronic lung infections by *P. aeruginosa* and *A. baumannii*, gallium nitrate has reduced lung injury and bacterial loads in tissues (Kaneko et al., 2007; de Léséleuc et al., 2012). Regarding SERM drugs, toremifene has shown efficacy against oral infection caused by *Porphyromonas gingivalis* (Gerits et al., 2017), and raloxifene attenuated *in vitro* and in *Caenorhabditis elegans* model the virulence of *P. aeruginosa* by binding to PhzB2 which is involved in the production of pyocyanin, a pigment related with the virulence of this pathogen (Ho Sui et al., 2012).

In the case of fungi, the activity of anticancer drugs has also been investigated. Floxuridine, at twice its half maximal inhibitory concentration (IC_50_) value, has exhibited fungicidal activity against *Exserohilum rostratum* reducing the hyphae-derived CFU (colony-forming unit) of this fungus (Sun et al., 2013). The SERM compounds such as tamoxifen and toremifene have also shown fungicidal activity against *C. neoformans*. They also display a number of pharmacological properties desirable for anticryptococcal drugs, including synergistic fungicidal activity with fluconazole and/or amphotericin B *in vitro* and *in vivo*, oral bioavailability, and activity within macrophages (Butts et al., 2014). They bind directly to the two essential EF-hand proteins calmodulin 1 (Cam1) and calmodulin-like protein 1 (Cml1) of *C. neoformans*, preventing Cam1 from binding to its well-characterized substrate calcineurin (Cna1), thereby blocking Cna1 activation (Butts et al., 2014). In whole cells, toremifene and tamoxifen are known to block the calcineurin-dependent nuclear localization of the transcription factor Crz1 (Butts et al., 2014). Together, both drugs have inhibited the growth of *C. neoformans* within macrophages, a niche not accessible to current antifungal drugs (Butts et al., 2014). In murine-disseminated cryptococcosis, tamoxifen in combination with fluconazole decreased the brain burden by ∼1 log_10_ CFU/g (Butts et al., 2014). Against *C. albicans* and *Candida glabrata*, toremifene has enhanced the antibiofilm activity of amphotericin B and caspofungin [fractional inhibitory concentration index (FICI) < 0.5 both *in vitro* and *in vivo* worm infection models (Delattin et al., 2014)].

Other anticancer drug such as finasteride, a 5-α-reductase inhibitor commonly used for the treatment of benign prostatic hyperplasia, was highly effective in both the prevention and destruction of *C. albicans* biofilm formation at doses greater than 16 and 128 mg/L, respectively (Chavez-Dozal et al., 2014). In combination with 2 mg/L fluconazole, 2 mg/L, finasteride exhibited synergistic activity in the prevention of biofilm formation by *C. albicans* (Chavez-Dozal et al., 2014). Similar effects were observed in the presence of doxorubicin and daunorubicin that inhibited the morphogenesis of *C. albicans* (Routh et al., 2013).

Anticancer drugs not only target bacteria and fungi but can also regulate the host response. Floxuridine and streptozotocin have presented a protective effect on the host by reducing *S. aureus*-mediated killing in human neutrophils (Yeo et al., 2018). Moreover, tamoxifen can stimulate chemotaxis, phagocytosis, and neutrophil extracellular trap (NET) formation through the modulation of the ceramide pathway upon infection with *S. aureus* (Corriden et al., 2015). Unlike tamoxifen, its analog raloxifene has been shown to reduce NET formation in human neutrophils, thus resulting in cell death of *S. aureus* (Flores et al., 2016). In addition, denileukin diftitox has been reported to bind to the IL-2 receptor in T lymphocytes, thereby introducing diphtheria toxin inside these cells to suppress them. The decrease in this type of T cell hinders the replication of *M. tuberculosis* (Gupta et al., 2017). It is noteworthy to mention that toxicity of anticancer drugs is important in terms of their establishment as antibacterial drugs. Tamoxifen has been used for over 30 years to treat breast cancer. The doses of tamoxifen used in animals (250 mg/kg) (Corriden et al., 2015) and in humans (20–40 mg) are generally tolerated. For clomiphene, acute oral LD_50_ in mice and rats were 1,700 and 5,750 mg/kg, respectively (Drug Bank, 2018). The toxic dose of clomiphene in humans is unknown but toxic effects accompanying acute overdosage were not observed (Drug Bank, 2018). In the case of gallium nitrate, the treatment of hypercalcemia was performed with continuous intravenous infusion (200 mg/m^2^/day during 5 days) being generally well tolerated (Warrell et al., 1988). On the other hand, higher doses (300 mg/m^2^/day during 5–7 days) were used in cancer and side effects such as diarrhea and renal toxicity were observed (Chitambar, 2010).

### Anti-inflammatory and Immunomodulatory Drugs Repurposed Against Bacteria and Fungi

As is the case with anticancer drugs, anti-inflammatory and immunomodulatory drugs have demonstrated more antibacterial activity against Gram-positive than Gram-negative bacteria and fungi.

Celecoxib, a non-steroidal anti-inflammatory drug (NSAID), showed antibacterial activity against several pathogens including *S. aureus, Bacillus anthracis, B. subtilis*, and *M. smegmatis* (Thangamani et al., 2015b). Celecoxib has demonstrated non-specific targeting by inhibiting bacterial DNA and RNA replication, protein synthesis, and cell wall formation (Thangamani et al., 2015b), as well as reducing the levels of IL-6, TNF-α, IL-1β, and MCP-1 (monocyte-chemoattractant protein-1) in skin lesions caused by *S. aureus* infection (Thangamani et al., 2015b). Moreover, this drug has exhibited synergy with several topical and systemic antimicrobials used against *S. aureus*, with the exception of linezolid (Thangamani et al., 2015b).

Other NSAIDs, such diflunisal in combination with diclofenac, ibuprofen and verapamil have shown antibacterial activity against *S. aureus* and *M. tuberculosis* (Dutta et al., 2007; Vilaplana et al., 2013; Gupta et al., 2015; Hendrix et al., 2016). It was reported that diflunisal did not affect the bacterial growth of *S. aureus in vitro*, but did inhibit their toxicity in murine and human osteoblasts *in vivo* (Hendrix et al., 2016). Confirmed data have been observed in mice treated with diflunisal, which have presented less cortical bone marrow destruction, although a reduction in the bacterial load was not observed (Hendrix et al., 2016). Even though bacterial growth was not compromised, diflunisal inhibited accessory gene regulator A (AgrA), a regulator protein which inhibits alpha-type phenol soluble modulins (PSMs) and may contribute to a reduction in *S. aureus* virulence (Hendrix et al., 2016). In the case of verapamil, it has potentiated the activity of bedaquiline, a novel drug used to treat MDR tuberculosis, against *M. tuberculosis* (Dutta et al., 2007; Gupta et al., 2015). Moreover, treatment with ibuprofen significantly decreased the bacterial load and increased mice survival in an experimental model of active tuberculosis (Vilaplana et al., 2013).

For Gram-negative bacteria, celecoxib and betamethasone in combination with antibiotics have demonstrated activity against different bacterial species (Artini et al., 2014; Thangamani et al., 2015b). Celecoxib has presented synergy with colistin against *A. baumannii, P. aeruginosa, Escherichia coli, K. pneumoniae* and *Salmonella enterica* serovar Typhimurium (Thangamani et al., 2015b), and betamethasone has demonstrated synergy with ceftazidime, erythromycin, and ofloxacin against *P. aeruginosa* and some strains of *E. coli* (Artini et al., 2014). Diclofenac, in turn, was found to exhibit efficacy both *in vitro* and *in vivo* against *S. enterica* serovar Typhimurium (Dutta et al., 2007). In the case of glatiramer acetate, a drug used in the treatment of multiple sclerosis, activity against *A. baumannii, P. aeruginosa*, and *E. coli* reference strains, and against *A. baumannii* and *P. aeruginosa* clinical isolates from bacteremia and chronic respiratory infections in cystic fibrosis patients has been observed by disruption of the biofilm formation (Christiansen et al., 2017).

Like anticancer drugs, some anti-inflammatory and immunosuppressive drugs such as aspirin, ibuprofen, and tacrolimus have shown antifungal activity against *C. neoformans, C. gattii*, and *E. rostratum*, respectively (Sun et al., 2013; Ogundeji et al., 2016). The treatment of cryptococcal cells with aspirin and ibuprofen has led to the induction of stress via activation of the high-osmolarity glycerol (HOG) pathway in *C. neoformans* and *C. gattii*, and to their death through the activation of reactive oxygen species (ROS)-mediated membrane damage (Ogundeji et al., 2016). The MICs of these drugs did not negatively affect growth or impair macrophage function; rather, they enhanced the ability of these immune cells to phagocytose cryptococcal cells (Ogundeji et al., 2016). Moreover, treatment with tacrolimus at twice its IC_50_ value significantly reduced the hyphae-derived CFU of *E. rostratum* (Sun et al., 2013).

### Antipsychotic and Antidepressant Drugs Repurposed Against Bacteria and Fungi

Trifluoperazine, an antipsychotic drug, has showed therapeutic efficacy in a murine model of *C. difficile* infection, presenting higher survival rates than those treated with vancomycin; a decrease in inflammation and edema was also observed compared with the infected group (Andersson et al., 2016). Furthermore, together with amoxapine, trifluoperazine in combination with vancomycin protected 80% and 100% of mice, respectively, from severe oral infection caused by *C. difficile* (Andersson et al., 2016). Rani Basu et al. (2005) reported that the combination of two different non-antimicrobial drugs, prochlorperazine and methdilazine, may present antibacterial activity against *S. aureus*.

For Gram-negative bacteria, pimozide, used for the treatment of severe Tourette’s syndrome and schizophrenia, has reduced *in vitro* the internalization of *S. enterica* serovar Typhimurium and *E. coli* by phagocytic cells (Lieberman and Higgins, 2009). Moreover, pimozide reduced the bacterial uptake and vacuolar escape of *Listeria monocytogenes* in bone marrow-derived macrophages, as well as the invasion and cell-to-cell spread of the bacteria during the infection of non-phagocytic cells (Lieberman and Higgins, 2009). In addition, the drugs trifluoperazine and amoxapine were shown to be active against *Yersinia pestis* after screening of a library of 780 FDA-approved drugs to identify molecules which reduce *Y. pestis* cytotoxicity in murine macrophages (Andersson et al., 2016). These two compounds exhibited therapeutic efficacy in a murine model of pneumonic plague by *Y. pestis*; although the treatment was less effective when administration of the drug was delayed (Andersson et al., 2017). However, their efficacy was improved when both compounds were administered in combination with levofloxacin (Andersson et al., 2017). In addition to this study, amoxapine was reported to present therapeutic efficacy in an experimental murine model of respiratory infection caused by *K*. *pneumoniae* (Andersson et al., 2017). Finally, azathioprine, an antidepressant drug used for the treatment of Crohn’s disease, has exhibited anti-biofilm activity against *P. aeruginosa* and *E. coli* through the inhibition of WspR (Antoniani et al., 2013). WspR is a diguanylate cyclase involved in the regulation of a signal molecule called cyclic-di-GMP (c-di-GMP) known as a regulator of the bacterial biofilm formation (Antoniani et al., 2013).

In the case of fungi, the antipsychotic drug bromperidol has exhibited synergy with various azoles against *C. albicans, C. glabrata*, and *Aspergillus terreus* (Holbrook et al., 2017). Bromperidol has demonstrated synergy with posaconazole and voriconazole, and partial synergy with itraconazole and ketoconazole against *C. albicans, C. glabrata*, and *A. terreus*, as demonstrated by checkerboard and time-kill assays (Holbrook et al., 2017). Moreover, bromperidol in combination with posaconazole and voriconazole, increased the disruption of biofilm formation by sessile cells of *C. albicans* induced by both azoles. Their sessile MICs were reduced from >32 to 0.5 mg/L (Holbrook et al., 2017).

### Other Drugs Repurposed Against Bacteria and Fungi

Other drugs with different modes of action and clinical indications have been evaluated as antibacterial agents. Auranofin, which is used for the treatment of rheumatoid arthritis, has shown in monotherapy greater activity against a wide range of Gram-positive bacteria including *S. pneumoniae, S. aureus, Enterococcus faecium, E. faecalis*, and *Streptococcus agalactiae* when compared with vancomycin or linezolid (Aguinagalde et al., 2015; Thangamani et al., 2016a,b). *In vivo*, auranofin and its analogs have demonstrated therapeutic efficacy in different experimental models such as MRSA septicemic infection, MRSA skin infection, MRSA implant infection model (a model involving mesh-associated biofilm), and MRSA intramuscular infection model (Aguinagalde et al., 2015; Thangamani et al., 2016a,b). Interestingly, auranofin has demonstrated synergy with the commonly used antibiotics such as ciprofloxacin, linezolid, and gentamicin against MRSA (Thangamani et al., 2016b). In order to improve the activity of auranofin, different analogs were synthetized and display improved antibacterial activity against *S. aureus* and *S. pneumoniae causing bacteremia* in murine model (Aguinagalde et al., 2015). The mode of action of auranofin against *S. aureus* has been deciphered using the macromolecular biosynthesis assay which showed that auranofin acts on the inhibition of DNA replication and protein synthesis, downregulating the toxin production (Thangamani et al., 2016a).

Ebselen; despite the fact that it is not an FDA-approved drug, it is being investigated in clinical trials for the treatment of bipolar disorder and ischemic stroke, has also been evaluated. Two studies have suggested that this compound exhibited antibacterial activity against *C. difficile in vitro* and *in vivo* by targeting glucosyltransferase domain (GTD) of *C. difficile* toxins (Peng et al., 2018), and against MRSA and vancomycin-resistant *S. aureus* (VRSA) with MIC values <1 mg/L (Thangamani et al., 2015c). Moreover, ebselen has also reduced the biofilm formation by *S. aureus* (Gi et al., 2014). Synergy between this drug and fusidic acid, retapamulin, mupirocin, and daptomycin against *S. aureus* strains was confirmed using a Bliss model (Thangamani et al., 2015c).

Besides these two drugs, the antihistaminic compounds terfenadine and its analogs were also investigated as potential antibacterial drugs. Terfenadine has showed reasonable activity against *S. aureus* (Perlmutter et al., 2014). In order to improve their activity, 84 derivatives were synthesized that have presented greater MIC values against *S. aureus* (1 mg/L) as well as activity against *E. faecium, E. faecalis*, and *M. tuberculosis* (Perlmutter et al., 2014).

In the case of statins, simvastatin, used in the treatment of atherosclerotic cardiovascular disease and hypercholesterolemia, has exhibited antibacterial activity in monotherapy against *M. tuberculosis* (Parihar et al., 2014; Skerry et al., 2014). It marginally reduced the bacterial load 4 and 8 weeks after infection with *M. tuberculosis* by aerosol exposure; presumably by reducing cholesterol synthesis due to the inhibition of HMG-CoA reductase within the phagosomal membrane. This process has consequently enhanced the maturation of phagosomes, known to provide better defense against *M. tuberculosis*, and by inducing the autophagy of *M. tuberculosis*-infected macrophages (Parihar et al., 2014). The addition of simvastatin to the first-line tuberculosis therapy (rifampicin + isoniazid + pyrazinamide) may help to reduce mycobacterial infection and tissue damage in *M. tuberculosis*-infected mice (Skerry et al., 2014). Similarly, atorvastatin, another statin drug, has also demonstrated synergistic activity with rifampin *in vitro* against *M. tuberculosis* and in a murine model of *Mycobacterium leprae* infection (Lobato et al., 2014).

Regarding Gram-negative bacteria, auranofin exhibited synergy with polymyxin B against *A. baumannii, P. aeruginosa, K. pneumoniae* and *S. enterica* serovar Typhimurium (Thangamani et al., 2016a).

Ebselen has also presented antibacterial effect against *A. baumannii* and *E. coli* by reducing their bacterial growth at MICs of 32 μM and <128 μM, respectively. This bacterial reduction growth was due to the inhibition of TonB-mediated physiology, which is involved in iron acquisition from host sources (Nairn et al., 2017). Furthermore, ebselen exhibited anti-virulence activity against *P. aeruginosa* by targeting c-di-GMP signaling pathway, which regulates motility and biofilm formation (Gi et al., 2014; Lieberman et al., 2014).

In the case of statins, the combination of simvastatin with sub-inhibitory concentrations of colistin presented synergistic effect against a collection of *A. baumannii, E. coli, K. pneumoniae, P. aeruginosa*, and *S. enterica* serovar Typhimurium reducing the MIC of simvastatin from >256 mg/L to a range between 8 and 32 mg/L (Thangamani et al., 2015a). In addition, screening of an FDA-approved drug library has identified pentetic acid, an iron chelator, as an inhibitor of elastase, an important exoprotease as well as a reducer of biofilm formation by *P. aeruginosa* (Gi et al., 2014). When applied to *P. aeruginosa* infections in human lung tissue, pentetic acid increased the viability of human lung epithelial A549 cells post-infection (Gi et al., 2014). Interestingly, pentetic acid has also demonstrated therapeutic efficacy in a murine experimental model of respiratory infection by *P. aeruginosa* by increasing 42% the mice survival 5 days post-infection (Gi et al., 2014).

Moreover, calcitriol, a bioactive form of vitamin D3 used to treat hypocalcemic conditions and renal osteodystrophy, has been described as an enhancer of bactericidal activity against *P. aeruginosa*, due to its capacity to modulate the activity of monocytes and macrophages by increasing their bacterial killing (Nouari et al., 2015).

Other drugs that have presented anti-virulence effect against *P. aeruginosa* have been reported. For example 5-fluorocytosine, an antifungal drug, has been shown to reduce *in vitro* the production of virulence factors by *P. aeruginosa* such as pyoverdine, PrpL protease, and exotoxin A by downregulating *pvdS* gene expression (Imperi et al., 2013a), and to suppress *in vivo* the pathogenicity of *P. aeruginosa* in a murine model of lung infection (Imperi et al., 2013a). Other antifungal drugs such as clotrimazole and miconazole were identified as inhibitors of 2-heptyl-3-hydroxy-4 quinolone (PQS) quorum sensing (QS) system. This system is based on signal 2-alkyl-4-quinolones (AQ): PQS and 2-heptyl-4-hydroxyquinolone (HHQ) which can bind and activate the regulator PqsR and controls the expression of *P. aeruginosa* virulence factors. D’Angelo et al. (2018) have shown that probably both drugs modify PqsR function by competing with PQS and HHQ for the PqsR ligand-binding site. Finally, clofoctol and azithromycin, drugs originally developed as antibiotics against Gram-positive and Gram-negative bacteria, respectively, were found to have also anti-virulence properties against *P. aeruginosa* (Imperi et al., 2014; D’Angelo et al., 2018).

In the case of fungi, atorvastatin has demonstrated different effects on the host and the yeast by: (i) reducing the ergosterol content in the cell membrane and altering the properties of the polysaccharide capsule of *C. gattii*; (ii) increasing the production of ROS by macrophages; and (iii) reducing yeast phagocytosis and the intracellular proliferation rate (Ribeiro et al., 2017). Atorvastatin in combination with fluconazole was also tested as an adjuvant to control fungal infections. This combination demonstrated synergy *in vitro* against one strain of *C. gattii*. *In vivo*, atorvastatin plus fluconazole increased the survival of mice and reduced the burden of *C. gattii* in the lungs and brain (Ribeiro et al., 2017). Moreover, preclinical antimalarial drugs such as MMV665943 have been shown to inhibit and delay growth at submicromolar concentrations and exhibit fungicidal activity at concentrations greater than 1.56 μM against *C. albicans, C. neoformans, C. gattii* and *Lomentospora prolificans*. More specifically, this compound at concentrations greater than 1.56 μM affects the polysaccharide capsule thickness of *C. neoformans* (Jung et al., 2018).

Regarding the immune response modulation, ebselen and auranofin reduced the production of inflammatory cytokines such as TNF-α, IL-6, IL-1β, and MCP-1 in skin lesions infected by *S. aureus* (Thangamani et al., 2016a, 2015c). Similarly, calcitriol has shown a modulatory effect on monocytes and macrophages against *P. aeruginosa* infection by increasing their bacterial killing (Nouari et al., 2015). The mechanism of action of calcitriol on the immune system is unknown; although its downregulating effect on IL-1β, IL-6, and IL-8 has been observed (Xue et al., 2002). In the case of statin, simvastatin has been reported to modulate the production of proinflammatory cytokines (IL-8 and CCL20) and Kruppel-like factors (an emerging group of immune system regulators) in *P. aeruginosa* respiratory infections (Hennessy et al., 2014).

## Clinical Application of Repurposed Drugs Against Infectious Agents

Even though repurposed drugs showed promising preclinical data, to our knowledge only three clinical studies have been performed or are currently underway.

A randomized study on the role of aspirin in tuberculous meningitis suggested that aspirin in combination with corticosteroids reduced the incidence of strokes and mortality (Misra et al., 2010). A similar study on the role of aspirin as an adjunct with steroids for the treatment of HIV-negative adults with tuberculous meningitis in Vietnam is still ongoing, now in Phase II trial (clinical trials identifier: NCT02237365). Another Phase III trial (ClinicalTrials.gov Identifier: NCT02060006) is being conducted to evaluate the feasibility and efficacy of using meloxicam, a cheap and widely available NSAID, as a preventive intervention for tuberculous-immune reconstituted inflammatory syndrome; results from this study have yet to be published (Maitra et al., 2016).

## Conclusion and Perspectives

In the last decade, substantial progress has been made in the development of repurposed drugs for the treatment of bacterial and fungal infections. Several compounds have yielded promising data but developmental efforts remain in the preclinical stage. Additional relevant issues should be take into account in the preclinical development of repurposing drugs including possible need for new formulations to increase their bioavailability and ADMET tests if the administration route is changed, possible negative effect of the primary drug activity (especially for anticancer and antipsychotic drugs), and challenges for intellectual property rights. Moreover, further clinical studies are needed to address the urgent demand for new treatments targeting infections caused by bacteria and fungi.

## Author Contributions

AM-C, RA-A, and YS wrote the manuscript. All authors read and approved the final manuscript.

## Conflict of Interest Statement

The authors declare that the research was conducted in the absence of any commercial or financial relationships that could be construed as a potential conflict of interest.
