# Corrigendum: Drug Repurposing for the Treatment of Bacterial and Fungal Infections

**DOI:** 10.3389/fmicb.2022.844615

**Published:** 2022-02-25

**Authors:** Andrea Miró-Canturri, Rafael Ayerbe-Algaba, Younes Smani

**Affiliations:** Clinical Unit of Infectious Diseases, Microbiology and Preventive Medicine, Institute of Biomedicine of Seville (IBiS), University Hospital Virgen del Rocío, CSIC, University of Seville, Seville, Spain

**Keywords:** repurposing drug, bacteria, fungi, antimicrobial resistance, infection

There is an error in the Funding statement. The funder details for Grant No. PI16/01378 were incomplete. The corrected Funding statement appears below:

“This study was supported by Miguel Servet Tipo I Project grant, Instituto de Salud Carlos III, Subdirección General de Redes y Centros de Investigación Cooperativa, Ministerio de Economía, Industria y Competitividad (CP15/00132), the Instituto de Salud Carlos III, Proyectos de Investigación en Salud (Grant No. PI16/01378)—cofinanced by the European Development Regional Fund A way to achieve Europe, operative program Intelligent Growth 2014-2020. YS is supported by the Subprograma Miguel Servet Tipo I from the Ministerio de Economía y Competitividad of Spain (CP15/00132).”

The authors apologize for this error and state that this does not change the scientific conclusions of the article in any way. The original article has been updated.

## Publisher's Note

All claims expressed in this article are solely those of the authors and do not necessarily represent those of their affiliated organizations, or those of the publisher, the editors and the reviewers. Any product that may be evaluated in this article, or claim that may be made by its manufacturer, is not guaranteed or endorsed by the publisher.

